# Selective and Irreversible Inhibitors of Aphid Acetylcholinesterases: Steps Toward Human-Safe Insecticides

**DOI:** 10.1371/journal.pone.0004349

**Published:** 2009-02-04

**Authors:** Yuan-Ping Pang, Sanjay K. Singh, Yang Gao, T. Leon Lassiter, Rajesh K. Mishra, Kun Yan Zhu, Stephen Brimijoin

**Affiliations:** 1 Molecular Pharmacology and Experimental Therapeutics, Mayo Clinic, Rochester, Minnesota, United States of America; 2 Department of Entomology, Kansas State University, Manhattan, Kansas, United States of America; The Scripps Research Institute, United States of America

## Abstract

Aphids, among the most destructive insects to world agriculture, are mainly controlled by organophosphate insecticides that disable the catalytic serine residue of acetylcholinesterase (AChE). Because these agents also affect vertebrate AChEs, they are toxic to non-target species including humans and birds. We previously reported that a cysteine residue (Cys), found at the AChE active site in aphids and other insects but not mammals, might serve as a target for insect-selective pesticides. However, aphids have two different AChEs (termed AP and AO), and only AP-AChE carries the unique Cys. The absence of the active-site Cys in AO-AChE might raise concerns about the utility of targeting that residue. Herein we report the development of a methanethiosulfonate-containing small molecule that, at 6.0 µM, irreversibly inhibits 99% of all AChE activity extracted from the greenbug aphid (*Schizaphis graminum*) without any measurable inhibition of the human AChE. Reactivation studies using β-mercaptoethanol confirm that the irreversible inhibition resulted from the conjugation of the inhibitor to the unique Cys. These results suggest that AO-AChE does not contribute significantly to the overall AChE activity in aphids, thus offering new insight into the relative functional importance of the two insect AChEs. More importantly, by demonstrating that the Cys-targeting inhibitor can abolish AChE activity in aphids, we can conclude that the unique Cys may be a viable target for species-selective agents to control aphids without causing human toxicity and resistance problems.

## Introduction

Aphids are among the world's most destructive insect pests of grain crops, vegetables, ornamental plants, and fruit trees. For 150 years the greenbug aphid (*Schizaphis graminum*) has been a major pest of small grains (e.g., sorghum and wheat). Annual costs for greenbug control in wheat production have been estimated at up to $100 million on the Texas High Plains alone [1]. The soybean aphid (*Aphis glycines*) causes combined US yield losses and increased production costs that exceed $1 billion [2]. Aphid control relies mainly on a small number of highly toxic anticholinesterases approved by the US Environmental Protection Agency (EPA); the threat to agriculture and environmental health is growing. This phenomenon is partly due to an unusual feature of aphid biology. During the aphid-growing season all aphids become female, and are able to produce offspring by maternal cloning in a process known as parthenogenesis. This form of reproduction, with up to 18 asexual generations per growing season [3], allows aphids to develop resistance rapidly when few effective insecticides are applied repeatedly, as often happens in crops such as soybeans.

Acetylcholinesterase (AChE, EC 3.1.1.7) is a serine hydrolase vital for regulating the neurotransmitter acetylcholine in mammals, birds, and insects [4]. Current anticholinesterase insecticides such as chlorpyrifos and methamidophos phosphorylate a serine residue at the active site of AChE, thus disabling its function and causing incapacitation. Because this serine residue is also present in mammalian and avian AChEs, use of these insecticides poses serious risks of toxicity to mammals, birds, and beneficial insects such as the honeybee [5]. The US EPA has concluded that such agents can enter the brain of fetuses and young children and may damage the developing nervous system [6]. Controlling aphids in a large field requires insecticides at quantities toxic to mammals and birds. Unintended environmental toxicity is a concern associated with current agents used to manage these insects. In light of this concern, and the problem of insecticide resistance described above, there is an urgent need for new agents that are both safer and more effective in controlling aphids and related pests.

**Figure 1:**
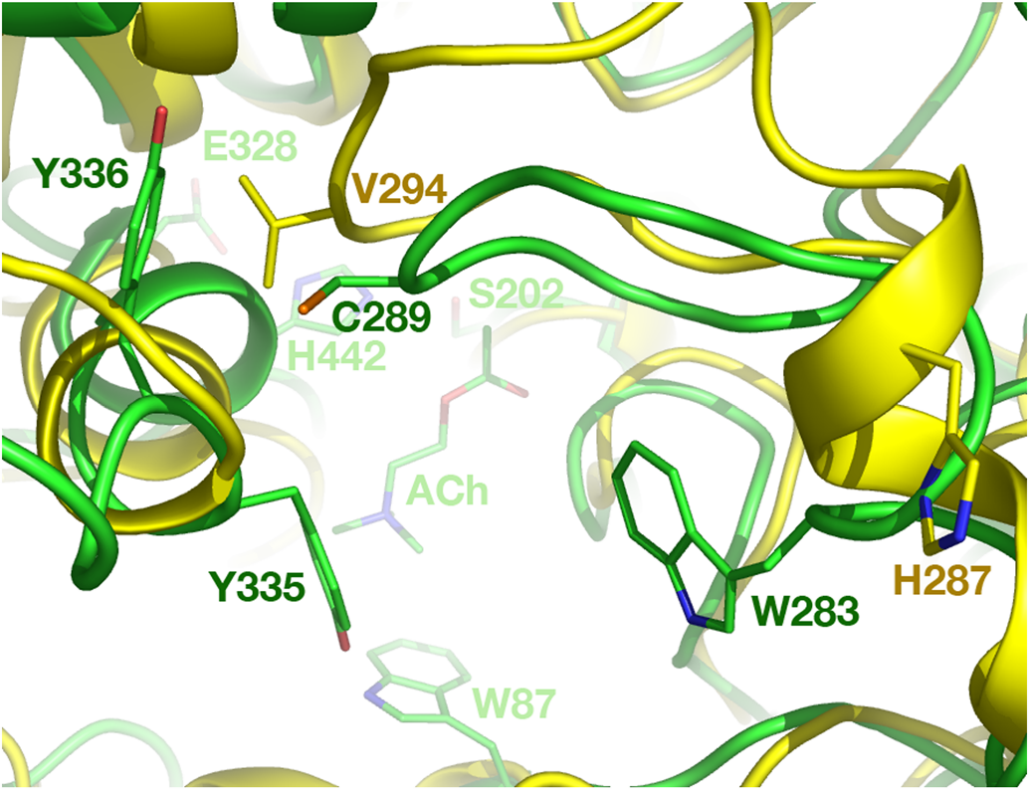
Top view of an overlay of three-dimensional structures of the greenbug and human acetylcholinesterase from a perspective looking down onto substrate acetylcholine at the catalytic site. The C, N, O, and S atoms are colored in yellow/green (human/greenbug), blue, red, and orange, respectively. The theoretical model of the greenbug enzyme and the crystal structure of the human enzyme were obtained from the coordinate files with Protein Data Bank codes of 2HCP and 1B41, respectively.

**Figure 2:**
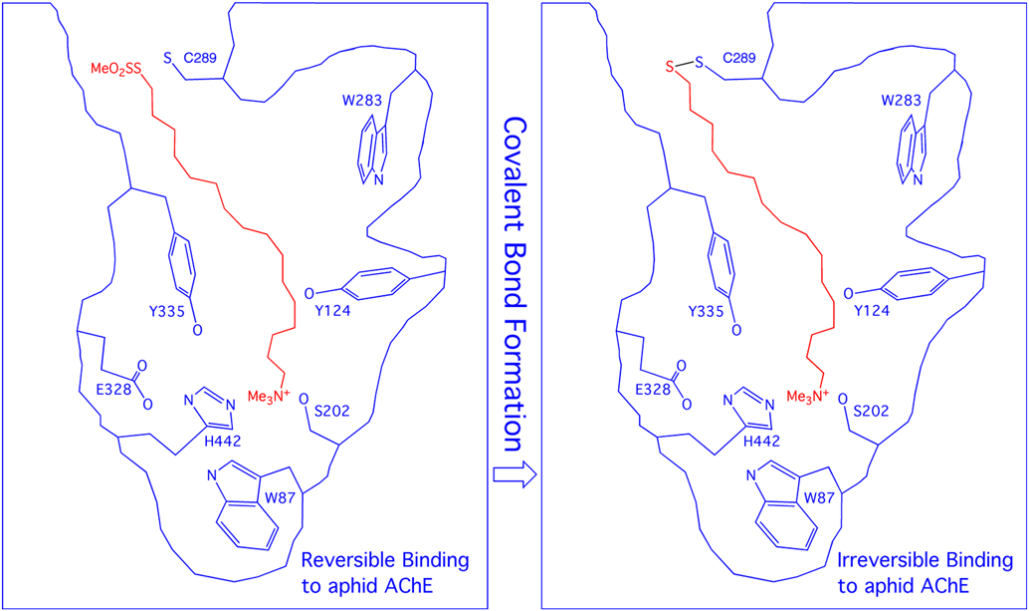
Diagram representation of a methanethiosulfonate-bearing inhibitor (*red*) that reacts with Cys289 of the greenbug acetylcholinesterase (*blue*) upon its binding at the active site.

A new concept for insect control is to use an irreversible inhibitor that targets an insect-specific region of an essential protein in the target species. Sequence analyses of various insect proteins identified a cysteine residue that is absent in mammalian [7]–[9] and avian [8], [10] AChEs but conserved in the AChEs of aphids and several other insects [7]–[9], [11]. This sequence-based finding was consistent with the reports that aphid AChEs were sensitive to sulfhydryl inhibitors [12]–[16]. The sequence analysis [7] along with the site-directed mutagenesis and molecular modeling studies on an AChE from amphioxus (*viz.*, an elongated marine invertebrate) led to speculations (1) that the cysteine residue conserved in the aphid AChE is located near the top of the active-site gorge and sensitive to sulfhydryl inhibitors and (2) that high affinity bi-functional cholinergic reagents that react transiently with the active site serine and irreversibly with the cysteine residue could be candidates for selective aphicides [7]. The three-dimensional (3D) models of AChEs in the greenbug and the English grain aphid (*Sitobion avenae*) generated by using terascale computing were reported subsequently [9]. These models reveal that the unique cysteine residue (Cys289 of the greenbug AChE or its equivalent in the English grain aphid) is located at the entrance of the AChE active site [9]. In the human AChE crystal structure [17], the residue spatially corresponding to Cys289 is Val294 (Figure 1). Furthermore, according to the 3D models, Cys289 has a favorable sulfur-aromatic interaction [18] with Tyr336 and is accessible for covalent bonding to small molecules that bind at the active site (Figure 2).

In general, a native or engineered cysteine residue near or at the active site of an enzyme can “hook” (covalently bond to) a small molecule that binds, even loosely, at the active site, as long as that molecule carries a sulfhydryl moiety [19] or a leaving group that is vulnerable to the attack by the thiol group [20]. Thus, a cysteine proteinase can be inhibited selectively and irreversibly by a chemically stable molecule via “hook chemistry,” namely, an inhibitor binds near the cysteine residue and then forms an adduct with that residue [20]. Worth noting here, sulfhydryl reagents, including homologs [21] of the new irreversible methanethiosulfonate-containing inhibitors disclosed in this article, reportedly form adducts with a cysteine residue at the peripheral site of a mammalian AChE engineered with a His287Cys mutation, thereby interfering with substrate binding and catalytic activity [21], [22]. In fact, the alpha carbon atom of His287Cys in the human AChE is 11 Å away from that of Cys289 in the greenbug AChE that is superimposed onto the human enzyme (see Figure 1). Thus it is not an exact model for the insect case. However, these findings support the general principle that a free cysteine at the entrance of the AChE active site could be a suitable target. Below we describe evidence for adducts with such a target in the native greenbug AChE.

In this context, it appeared promising to use Cys289 or its equivalent in other aphid AChEs as a novel target site for insecticide development (Figure 2) [7]–[9], [11]–[16], [21], [22]. Inhibitors that target Cys289 should be less toxic to mammals than current anticholinesterases, which target the ubiquitous catalytic serine residue of all AChEs. Targeting Cys289 may alleviate resistance problems with current insecticides for two reasons. First, aphids and other insects have had no opportunity to develop resistance to Cys289-targeting insecticides as they have done with the serine-targeting agents that have been used for decades. Second, aphids may find Cys289 indispensable even under selective pressure because it stabilizes the conformations of key aromatic residues in AChE. Indeed, sequence analysis shows that the AChEs of green peach aphids (*Myzus persicae*) and cotton/melon aphids (*Aphis gossypii*) carry the equivalent of Cys289 [8], [9], although both aphids are resistant to many current insecticides.

The fruit fly (*Drosophila melanogaster*), long used as a model insect, has only one AChE gene [23]. Point mutations conferring insecticide resistance in this gene have been identified [24]. However, in anticholinesterase-resistant strains of the house mosquito (*Culex pipiens*) [25], no mutations were found in the gene orthologous to the one in *D. melanogaster*, termed AO-AChE, despite biochemical evidence of decreased AChE sensitivity to current insecticides [26], [27]. The inability to identify resistance-conferring mutations in AO-AChE led to the two-AChE-gene hypothesis that resistance-conferring mutations occur in an unidentified gene, termed AP-AChE, that is paralogous to the one in *D. melanogaster* [28]. This hypothesis was confirmed by the discovery of the AP-AChE genes in the greenbug [11] and subsequently in the malaria-carrying African mosquito (*Anopheles gambiae*) [29]. Further studies suggested that AP-AChE is the predominant form of AChE, expressed in the greenbug [28], diamondback moth (*Plutella xylostella*) [30], human lice (*Pediculus humanus*) [25], and insecticide-resistant mosquitoes (*Culex tritaeniorhynchus*) [31].

Informed by this background, our previous sequence analysis of the two AChE genes in insects showed that Cys289 in the greenbug AP-AChE gene or its equivalent in other AP-AChE genes is conserved in 16 insect species including aphids, but is missing in the corresponding AO-AChE genes (see Figure S2 of reference [9]). The reported preponderance of AP-AChE over AO-AChE [25], [28], [30], [31] supports the notion of Cys289 as a target site for novel insecticides. However, an inhibitor selective for one AChE gene might not be able to abolish all AChE activity in a given insect. To address this concern while experimentally testing the hypothesis that Cys-targeting compounds can be selective for insect AChEs, we synthesized a series of methanethiosulfonate-bearing inhibitors designed to have affinity for the AChE active site and preferential reactivity with Cys289 or its equivalents in insect AChEs. These agents were then compared in terms of their ability to irreversibly inhibit AChE activity in extracts of the greenbug and washed membranes from human red blood cells.

In this article, we report the development and initial characterization of these inhibitors. Without precedent, one of these, at 6.0 µM, caused 99% irreversible inhibition of total extractable greenbug AChE activity while showing neither reversible nor irreversible inhibition of the human AChE under the same assay conditions. Below we discuss the implications of these findings with regard to the functions of the two different AChEs in insects and the prospects for design of species-selective insecticides.

## Results

### Design of Selective and Irreversible Aphid AChE Inhibitors

**Figure 3:**
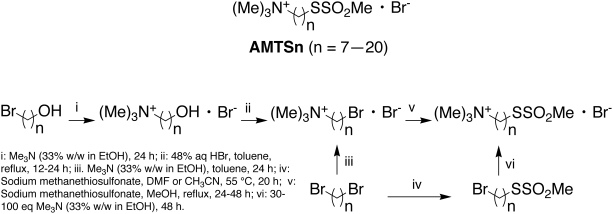
Chemical structures and synthetic schemes for AMTS7–AMTS20 as irreversible inhibitors of the greenbug acetylcholinesterase.

**Figure 4:**
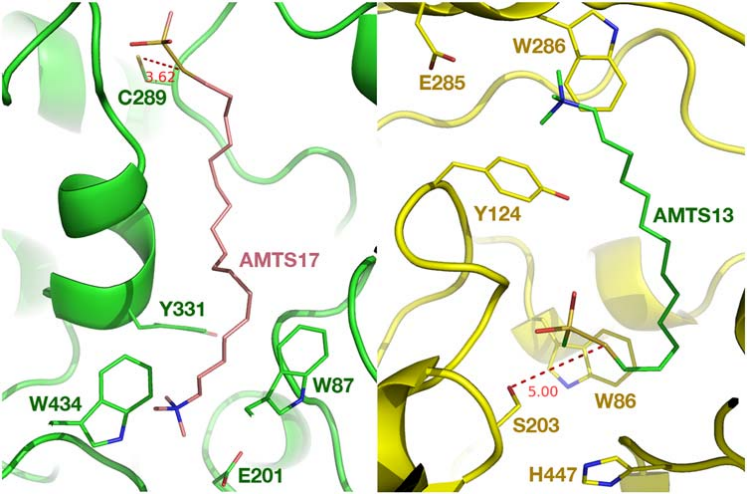
Cross-section view of the multiple-molecular-dynamics-simulation-refined three-dimensional models of the greenbug and human acetylcholinesterases in complex with AMTS17 and AMTS13, respectively. *Left*: The C, N, O, and S atoms are colored in magenta/green (AMTS17/AChE), blue, red, and yellow, respectively. *Right*: The C, N, O, and S atoms are colored in green/yellow (AMTS13/AChE), blue, red, and yellow, respectively.

To experimentally test the hypothesis that a species-specific cysteine residue in aphids could be a target for novel insecticides, we made a series of prototypical irreversible inhibitors (**AMTS7**–**AMTS20**) shown in Figure 3. Inspired by reference [21], we designed these inhibitors with (1) affinity for the greenbug AChE active site in order to build up their local concentration around Cys289 and (2) preferential reactivity to form an adduct with Cys289 or its equivalent, at the entrance to the active site, after binding near that locus (Figure 2). Therefore, all inhibitors of the series contained (1) a trimethylammonium group to confer affinity by the cation-pi interaction with Trp87 at the active site [32], [33] and (2) a methanethiosulfonate group known to form an adduct preferentially with a free cysteine residue [34]. As an added design feature, a chain of variable length separated the trimethylammonium group from the reactive methanethiosulfonate group as a means of controlling the access of the reactive group to Cys289 or other nucleophiles. Our multiple 10-ns-long molecular dynamics simulations of the greenbug AChE in complex with **AMTS17** suggested that the 17-methylene-long inhibitor would tuck into the active site with (1) its thiol sulfur atom located 3.6-Å away from the sulfur atom of Cys289 at the opening of the active site, while (2) its ammonium group engages in an ionic interaction with Glu201 as well as cation-pi interactions with Trp87, Tyr331, and Trp434 at the bottom of the active site (Figure 4a).

### Detecting Irreversible AChE Inhibition

Because only one of the two aphid AChEs carries a cysteine residue at the entrance of the active site [8], [9], [11], [28], the utility of our proposed hook chemistry depended upon the percentage of enzyme activity that could be irreversibly inhibited by the sulfhydryl reagents. To measure this variable, we developed an approach in which the total AChE-containing homogenate of insect or mammalian samples was exposed to a candidate inhibitor for a defined period of time, after which the unbound inhibitor was removed from AChE by extended dialysis or centrifuge-spin separation through a gel-filtration size-exclusion column (see Methods, “Measurement of AChE inhibition”). Assays of AChE activity in the inhibitor-containing and inhibitor-free preparations, when compared with a control, allowed us to determine the levels of total and irreversible AChE inhibition, respectively. The assays were performed under conditions that permitted accurate determinations on sub-milligram samples (less than a single aphid), using a radiometric method that was not influenced by free thiol groups in samples or reagents.

### Selective and Irreversible Inhibition of Aphid AChE

**Figure 5:**
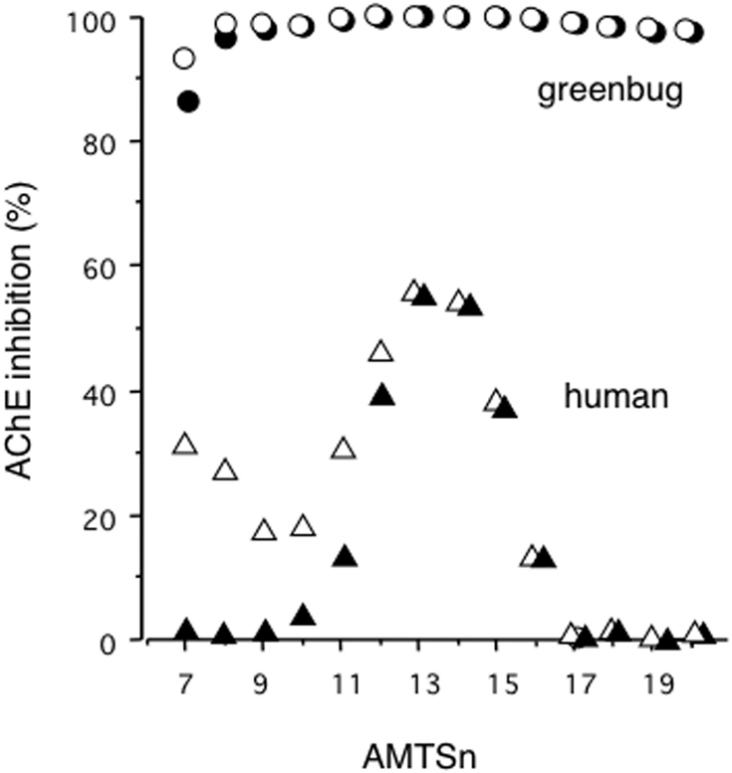
Inhibition of the greenbug and human AChEs by AMTS7–AMTS20. Extracts of greenbugs and human RBCs in 0.1 M sodium phosphate buffer (see Methods) were exposed to AMTS7–AMTS20 at 6.0 µM for one hour at room temperature. Radiometric assays were used to determine AChE activity in aliquots of treated samples either immediately (empty symbols) or after overnight dialysis with 2 changes of buffer in 100-fold excess (filled symbols). The difference between the paired measurements (i.e., the recovery after dialysis) indicates reversible inhibition.

**AMTS7**–**AMTS20** in concentrations from 1–100 µM were tested on insect and human samples. One-hour exposures of these compounds at a final concentration of 6.0 µM produced varying AChE inhibition in extracts from the greenbug and human red blood cells (RBCs). As expected, all the tested compounds irreversibly inhibited 87–99% of the AChE activity in greenbug extracts (Figure 5). In RBC extracts, by contrast, the short-chain inhibitors (**AMTS7**–**AMTS9**) caused weak and purely reversible inhibition at the same concentration. Somewhat surprisingly, exposing the human enzyme to the mid-length inhibitors (**AMTS10**–**AMTS16**) led to a degree of apparently irreversible inhibition. **AMTS13** was the most potent agent in this respect, and hence least desirable. In one series of experiments, which produced the data shown in Table 1, **AMTS13** at 6.0 µM led to 43% inhibition after 1 hr, 70% after 6 hr, and 78% after 16 hr. Gratifyingly, however, the long-chain inhibitors (**AMTS17**–**AMTS20**) showed unprecedented AChE species-selectivity, causing only 0 to 2% inhibition of the RBC AChE (Figure 5) but 99% inhibition of the greenbug enzyme.

**Table 1:** Reactivation Studies of the Greenbug and Human Acetylcholinesterases (AChEs) Inhibited by **AMTS13** Using β-Mercaptoethanol (BME) That Can Reverse the **AMTS13** Inhibition and Concomitantly Denature the Disulfide-Containing AChEs.

Sample	Pretreatment	Treatment	% AChE activity	% AChE activity reduced by BME and/or AMTS13	% AChE activity recovered by BME
Greenbugs	None	None	100	0	
	”	BME 0.5 hr	36	64	
	”	BME 1 hr	31	69	
	”	BME 2 hr	21	79	
	**AMTS13**	None	1	99	0
	”	BME 0.5 hr	12	88	33
	”	BME 1 hr	17	83	55
	”	BME 2 hr	16	84	76
RBCs	None	None	100	0	
	”	BME 0.5 hr	49	51	
	”	BME 1 hr	44	56	
	”	BME 2 hr	35	65	
	**AMTS13**	None	57	43	0
	”	BME 0.5 hr	28	72	0
	”	BME 1 hr	26	74	0
	”	BME 2 hr	20	80	0

The greenbug and RBC extracts were exposed to **AMTS13** (6.0 µM) for 1 hr and/or to BME (100.0 mM) for different periods of time. After the exposure(s), samples were dialyzed overnight and the AChE activity was measured. Activities are mean values of triplicate determinations expressed as percentages of the AChE activity.

### Target for Irreversible Aphid AChE Inhibition

To confirm that the methanethiosulfonate-induced inhibition was truly irreversible and not pseudo-irreversible (*i.e.*, caused by extremely slow dissociation), we studied a nonselective inhibitor (**AMTS13**) and a selective inhibitor (**AMTS18**) in more detail. For that purpose enzyme extracts of RBCs and the greenbug were treated with **AMTS13** or **AMTS18** for 1 hour and then dialyzed 24, 72, and 200 hours with twice-daily changes of buffer (sodium phosphate 0.1 M, pH 7.4). When AChE activity was subsequently tested there was no time-dependent recovery of the greenbug enzyme at any point. The effects of **AMTS13** on both enzymes were also stable over time. That is, the inhibitions of the greenbug and RBC AChEs by **AMTS13** remained at 99±0.6% and 43±4%, respectively. Hence, with both enzymes the inhibition was truly irreversible.

Subsequently, to investigate the nature of potential adducts that caused the irreversible inhibition and their involvement of cysteine residues in AChE, we performed reactivation experiments with different concentrations of β-mercaptoethanol (BME) [34]. All the following **AMTS13** treatments involved prior inactivation by **AMTS13** (6.0 µM, 1 hr) followed by overnight dialysis. Low concentrations of BME (1–10 mM) reactivated the greenbug AChE weakly after an **AMTS13** treatment (data not shown), but 100 mM BME was more effective in reactivation even though it caused a minor loss of the AChE activity on its own. When fresh greenbug and red cell extracts were treated for 0.5, 1.0, and 2.0 hours with 100 mM BME and then dialyzed (see Methods), the human AChE activity fell to 36%, 31%, and 21% of the control, respectively (Table 1). However, the greenbug AChE pretreated with **AMTS13** was dramatically reactivated by a two-hour exposure to 100 mM BME. In fact, the enzyme recovered from essentially zero activity (99% inhibition) to a level that was 76% of that in companion samples exposed only to BME (Table 1). In contrast, the human AChE pretreated with **AMTS13** did not recover any enzymatic activity after the same BME treatment. Instead, BME depressed the AChE activity of those samples even further and substantially. These results confirm that the inhibition of the greenbug AChE by **AMTS13** was truly irreversible and resulted from the conjugation of the [(Me)_3_N^+^(CH_2_)_13_S−] fragment of **AMTS13** to the insect-specific Cys in the active site of AP-AChE. The results also indicate that the partial irreversible inhibition of the human AChE by the undesirable nonselective **AMTS13** involved a different mechanism, the exploration of which lies beyond the focus of this paper. It is conceivable that the long-chain inhibitors (**AMTS17**–**AMTS20**) are selective and irreversible inhibitors of the aphid AChE that can pave the way toward development of safer and more effective insecticides for aphid control.

## Discussion

### Selective and Irreversible Inhibition of Aphid AChE

The present results provide direct experimental support for our previously published hypothesis that targeting the insect-specific cysteine residue can lead to safer and more effective insecticides and thereby serve as the basis for production of species-selective insecticides [8], [9]. The long-chain inhibitors (**AMTS17**–**AMTS20**) we developed to date achieved near total and essentially permanent inhibition of the greenbug AChE at micromolar concentrations of exposure while, under identical conditions, they scarcely affected the corresponding enzyme in humans. Furthermore, our preliminary studies show that the long-chain inhibitors also exhibited selective irreversible inhibition of total AChE activity of soybean aphids (*Aphis glycines*) at an inhibitor concentration of 6.0 µM (data not shown).

It is worth noting, however, these inhibitors are *prototypes* that are not necessarily suitable for field application. As yet they have not been tested to determine the relationship between the effective inhibitory concentration and the reaction time as well as their toxicity at a chosen concentration to aphids or other target species, or to confirm their predicted safety for mammals and birds. Likewise, there is no information regarding the physical stability of these methanethiosulfonates under field conditions or their persistence in soil and groundwater. Nonetheless, we regard the *in vitro* demonstration of species selectivity and essentially permanent inhibition of insect AChEs by our prototypes as not only proof of concept but also an exceedingly promising beginning to search for conceptually new insecticides that will be useful in agriculture while posing less environmental risk than current insecticides.

### Insight into the Greenbug AO-AChE Function

The functional roles of insect AO-AChE and AP-AChE are still unclear, partly because it was not formerly possible to inactivate either gene product selectively. However, the results described above suggest that AP-AChE will prove to be functionally more important, at least in the greenbug. The AO-AChE of aphids does not carry a cysteine residue at the active site according to our reported sequence analysis of AChE genes in insects [8]. As demonstrated in the present study, **AMTS18** do not reversibly or irreversibly inhibit the human AChE, and hence these compounds should not irreversibly inactivate the aphid AO-AChE. In other words, these compounds are plausible selective and irreversible inhibitors of the aphid AP-AChE, and yet they irreversibly inactivated 99% of the total AChE activity in our greenbug extracts. We see two possible explanations for this observation: (1) AO-AChE is poorly extracted and not measured in our assay; (2) AO-AChE is a minor contributor to the total acetylcholine-hydrolysis activity in the greenbug. The first explanation appears unlikely for several reasons. First, our extraction conditions used extensive mechanical homogenization to create fine suspensions from greenbug samples, in which all of the AChE should have been accessible to substrate. Second, our assays were performed directly on the suspensions without first removing insoluble matter by centrifugation or filtration. And third, in preliminary experiments with the fruit fly, whose well-characterized genome includes *only* the active-site-cysteine-free AO-AChE [23], the identical extraction protocol rendered abundant fruit fly AChE activity that was resistant to **AMTS18**. Therefore, we infer that the greenbug AO-AChE is indeed resistant to **AMTS18** and, hence, that this enzyme form does not contribute significantly to the total acetylcholine-hydrolyzing activity in the greenbug.

### Species-Selective Insecticide Targets

After sequence analysis of AChEs in 73 species [8], [9] we reported that Cys289 or its equivalent is absent in mammalian AChE but is conserved in the AP-AChE gene of 16 insects: house mosquito, Japanese encephalitis mosquito (*Culex tritaeniorhynchus*), the malaria-carrying African mosquito, German cockroach (*Blattella germanica*), rice leaf beetle (*Oulema oryzae*), cotton bollworm (*Helicoverpa armigera*), beet armyworm (*Spodoptera exigua*), codling moth (*Cydia pomonella*), diamondback moth (*Plutella xylostella*), domestic silkworm (*Bombyx mori*), honeybee (*Apis mellifera*), bird cherry-oat aphid (*Rhopalosiphum padi*), greenbug, melon or cotton aphid, green peach aphid, and English grain aphid. Except for the honeybee and silkworm, all others are pests. We found that the recently determined AP-AChE of the yellow fever mosquito (*Aedes aegypti*) also carries the active-site cysteine residue (unpublished result). The AP-AChE genes from additional pests will likely be determined in the near future. This outcome should add impetus to the increasingly well-validated pesticide strategy of targeting insect-specific cysteine residue.

Cysteine-targeting inhibitors like those described here should be far superior to current anticholinesterases in their lack of (1) resistance currently established in insects and (2) harm to no-target organisms. However, like current anticholinesterases cysteine-targeting inhibitors pose potential risks to the honeybee and silkworm, which also carry the insect-specific cysteine residues. Fortunately, there are realistic prospects for designing inhibitors with greater specificity within this broad group of organisms. Targeting another insect-specific residue in addition to Cys289 or its equivalent might minimize toxicity to bees or silkworms. In fact, we recently identified a second residue as a possible species-specific target in insects. This residue, Arg339 of the malaria-carrying AP-AChE, is absent in mammals and many insects but conserved at the entrance of the AP-AChE active site in the malaria-carrying African mosquito, the house mosquito, the Japanese encephalitis mosquito, and the German cockroach [8]. Our new analysis shows that the unique arginine residue is conserved in the yellow fever mosquito AP-AChE as well. It is logical to expect that targeting both Cys286 and Arg339 of *A. gambiae* AP-AChE could lead to specific “mosquitocides” that spare mammals, birds, honeybees, silkworms, and other beneficial insects. As to the aphid control, the species-selective insecticide strategy described above can be extended to targeting a residue in aphid AChEs that adopts a unique conformation in the 3D aphid AChE structures (which we refer to as an “aphid-conformation” residue) in comparison to the same residue in AChEs of other species. Targeting both aphid-specific and aphid-conformation residues could avoid the toxicity to beneficial insects.

### Possible Reaction of AMTS13 with Activated Serine

The mechanism by which **AMTS13** causes partial but persistent inhibition of the human AChE is worth discussing, although it is not directly pertinent to our aim of developing selective and irreversible inhibitors of insect AChEs. Methanethiosulfonates are known to react preferentially with cysteine over other residues [34] and are widely used as probes to distinguish cysteine from serine [35]–[37]. On the other hand, there is no free cysteine residue in the active site of the human AChE. Six of the eight cysteine residues in human AChE [38] are oxidized to form intrasubunit disulfide bonds according to the crystal structure of the human AChE [17]; the remaining two are located at the *N*- and *C*-termini, respectively [38] but the *N*-terminal cysteine residue is removed during biosynthesis [39] while the *C*-terminal one forms an intersubunit disulfide bond [40]. The observation of 43% irreversible inhibition of the human AChE by **AMTS13** was therefore puzzling.

As apparent from Figure 5, the irreversible inhibition of the human AChE is far more sensitive to the chain lengths of the inhibitors than the corresponding inhibition of the greenbug enzyme; nearly two-fold more irreversible inhibition was observed with the insect enzyme than with the human enzyme at the same assay conditions. These results suggested that the residue that reacts with **AMTS13** in the human AChE was much less reactive than Cys298 in the greenbug AChE. A residue in the human AChE whose reactivity may fall in that category is Ser203, which is activated for nucleophilic reactions by nearby His447 and Glu334 through a network of hydrogen bonds [17], although methanethiosulfonates do not react with “ordinary” serine residues [34]–[37].

Our multiple 10-ns-long molecular dynamics simulations of the human AChE in complex with **AMTS13** suggest that the inhibitor is able to span the active site with (1) its thiol sulfur atom located 5.0-Å away from the Ser203 hydroxyl oxygen atom and (2) its ammonium group engaged in an ionic interaction with E285 and cation-pi interactions with Tyr124 and Trp286 (Figure 4b). Our model reaction of **AMTS13** with 34 equivalents of CD_3_ONa also showed instant formation of 13-[methoxythio(d_3_)]-*N*,*N*,*N*-trimethyltridecan-1-aminium bromide at room temperature. The resulting sulfenate ester (Me_3_N^+^(CH_2_)_13_-S-O-CD_3_) was found to be stable at room temperature under the basic condition, which is consistent with the report of aliphatic sulfenate esters [41]. These results support the hypothesis that **AMTS13** reacts with the catalytic serine residue in the human enzyme. Our simulations also revealed that the side-chain conformation of Trp86 in the human AChE bound with **AMTS13** is very different from the conformation in the free enzyme, implying a high-energy cost associated with the binding of **AMTS13**. This feature may account for the fact that only 43% of the human AChE was irreversibly inhibited by a one-hour exposure to **AMTS13**.

Structural studies with liquid chromatography mass spectrometry and/or crystallography analysis are needed to confirm the posited adduct of **AMTS13** to Ser203 in the human AChEs. Further studies are also needed to investigate the possibility that **AMTS13** may form micelles at 6.0 µM and partially inhibit human AChE through enzyme denaturation. Meanwhile, the present results do raise caution with regard to the use of methanethiosulfonate-based agents to probe cysteine residues in the presence of a serine residue that is activated for nucleophilic reactions.

## Materials and Methods

### Sources

Greenbugs were obtained from the Department of Entomology at Kansas State University. Soybean aphids were collected from demonstration plots at the University of Minnesota Extension facility in Rochester, Minnesota. Fruit flies were received from Dr. A. Tang in Transplant Biology at the Mayo Clinic. Group AB human red blood cells were obtained from Sigma-Aldrich (St Louis, MO) as were β-mercaptoethanol, and acetylcholine iodide. Tritiated acetylcholine (99 mCi/mM) was purchased from New England Nuclear (Waltham, MA). **AMTS7**–**AMTS20** were synthesized as described below.

### Chemical Syntheses

The ^1^H and ^13^C NMR spectra were recorded on a Varian Mercury 400 spectrometer. Chemical shifts are reported in ppm using either tetramethylsilane or the solvent peak as an internal standard. Data are reported as follows: chemical shift, multiplicity (s = singlet, d = doublet, t = triplet, q = quartet, m = multiplet), coupling constant and integration. Elemental analyses were performed by Atlantic Microlab, Inc. (Norcross, Georgia). Medium Pressure Liquid Chromatography (MPLC) was performed with Biotage SP-1 (Charlottesville, VA) using silica gel (EM Science, 230–400 mesh). Trimethylamine (33% w/w solution in ethanol), 1,8-dibromooctane, 1,10-dibromodecane, 1,11-dibromoundecane and 1,12-dibromododecane were purchased from Acros Organics (Geel, Belgium). 1,7-Dibromoheptane and 1,9-dibromononane were purchased from Aldrich Chemical Company, Inc. (Milwaukee, WI). 1,14-Dibromotetradecane was purchased from Pfaltz & Bauer, Inc. (Waterbury, CT). 13-Bromo-1-tridecanol, 15-bromo-1-pentadecanol and 16-bromo-1-hexadecanol were purchased from AstaTech, Inc. (Bristol, PA). Other 1,n-dibromoalkanes (where n = 17–20) [42], [43] and sodium methanethiosulfonate [44] were synthesized following literature procedures. 10-Bromo-*N*,*N*,*N*-trimethyldecan-1-aminium bromide [45], 12-bromo-*N*,*N*,*N*-trimethyldodecan-1-aminium bromide [45], 15-hydroxy-*N*,*N*,*N*-trimethylpentadecan-1-aminium bromide [46] and 16-hydroxy-*N*,*N*,*N*-trimethylhexadecan-1-aminium bromide [46] were synthesized following literature procedures.

### Procedure i: Synthesis of n-hydroxy-*N*,*N*,*N*-trimethylalkan-1-aminium bromide

Trimethylamine (33% w/w solution in ethanol, excess 20–100 eq) was added to n-bromoalkan-1-ol (1 eq) and left for 24 h. The solid obtained was collected by filtration, washed with hexanes/ethyl acetate mixtures (9/1, 3×3 mL/mmol) in a sonicator. The residue was filtered and dried to give n-hydroxy-*N*,*N*,*N*-trimethylalkan-1-aminium bromide.

### Procedure ii: Synthesis of n-bromo-*N*,*N*,*N*-trimethylalkan-1-aminium bromide

Hydrobromic acid (48% aqueous, 10 mL/mmol) and toluene (10 mL/mmol) were added to n-hydroxy-*N*,*N*,*N*-trimethylalkan-1-aminium bromide and refluxed using a Dean-Stark apparatus till no more water was collected (12–24 h). Toluene was removed under vacuum, and the residue was sonicated in a mixture of hexanes/ethyl acetate (9/1, 3×10 mL/mmol) and filtered to give n-bromo-*N*,*N*,*N*-trimethylalkan-1-aminium bromide.

### Procedure iii: Synthesis of n-bromo-*N*,*N*,*N*-trimethylalkan-1-aminium bromide

Trimethylamine (1 eq, 33% w/w solution in ethanol) was added dropwise to the 1,n-dibromoalkane (1.2 eq) in toluene (0.1 mL/mmol) and left aside in the dark for 48 h. The solid formed was collected by filtration and triturated with acetone (2×30 mL). The combined acetone layer was concentrated and dried to give n-bromo-*N*,*N*,*N*-trimethylalkan-1-aminium bromide.

### Procedure iv: Synthesis of *S*-n-bromoalkyl methanesulfonothioate

Sodium methanethiosulfonate (1 eq) was added to a clear solution of 1,n-dibromoalkane (5 eq) (longer chain analogues needed slight warming for complete dissolution) in dimethylformamide or acetonitrile (3 mL/mmol) and the mixture heated at 55°C for 20 h. The cooled reaction mixture was poured into water (3 mL/mmol) and extracted with ethyl acetate (3×3 mL/mmol). The combined organic layer was dried over anhydrous magnesium sulfate, concentrated under vacuum and refined by MPLC (gradient from 0 to 20% ethyl acetate in hexanes) to give *S*-n-bromoalkyl methanesulfonothioate.

### Procedure v: Synthesis of *N*,*N*,*N*-trimethyl-n-(methylsulfonylthio)alkan-1-aminium bromide

The n-bromo-*N*,*N*,*N*-trimethyl-*n*-alkan-1-aminium bromide (1 eq) and sodium methanethiosulfonate (0.95–1.10 eq) were refluxed in methanol (5–10 mL/mmol) for 24–48 h. The reaction mixture was cooled and concentrated under vacuum. The residue was sonicated in acetone. The insolubles (sodium bromide) were filtered and the filtrate was concentrated under vacuum. The concentrated filtrate was sonicated in hexanes (20 mL/mmol) and the solid collected by filtration to give the product, which was purified by recrystallization from acetone.

### Procedure vi: Synthesis of *N*,*N*,*N*-trimethyl-n-(methylsulfonylthio)alkan-1-aminium bromide

*S*-n-Bromoalkyl methanesulfonothioate (1 eq) was treated with excess trimethylamine (33% w/w solution in ethanol, 30–100 eq) and left aside for 48 h. The solution was concentrated under vacuum and the residue was sonicated in hexanes (20 mL/mmol) and the solid was collected by filtration to give the product, which was purified by recrystallization from acetone.

**7-Bromo-** ***N*** **,** ***N*** **,** ***N*** **-trimethylheptan-1-aminium bromide** (331 mg, 6%) was obtained from 1,7-dibromoheptane (5.14 g, 20 mmol) and trimethylamine (4 mL, 33% w/w solution in ethanol, 16.74 mmol) following procedure **iii**. ^1^H NMR (CDCl_3_): 3.62–3.58 (m, 2H), 3.42 (s, 9H), 3.38 (t, *J* = 6.6 Hz, 2H), 1.84–1.77 (m, 2H), 1.76–1.70 (m, 2H), and 1.44–1.31 (m, 6H); ^13^C NMR (CDCl_3_): 66.6, 53.3, 33.9, 32.3, 28.1, 27.6, 25.8, and 22.9.

***N*** **,** ***N*** **,** ***N*** **-Trimethyl-7-(methylsulfonylthio)heptan-1-aminium** (**AMTS7**, 174 mg, 19%) was obtained from sodium methanethiosulfonate (388 mg, 2.89 mmol) and 7-bromo-*N*,*N*,*N*-trimethylheptan-1-aminium bromide (830 mg, 2.62 mmol) following procedure **v**. ^1^H NMR (DMSO-*d*_6_): 3.53 (s, 3H), 3.31–3.27 (m, 2H), 3.21 (t, *J* = 7.2 Hz, 2H), 3.05 (s, 9H), 1.74–1.62 (m, 4H), 1.44–1.33 (m, 4H), and 1.31–1.22 (m, 2H); ^13^C NMR (DMSO-*d*_6_): 65.9, 52.8, 50.8, 36.1, 29.4, 28.5, 28.3, 26.2, and 22.6; Anal. Calcd (%) for C_11_H_26_NO_2_S_2_ Br: C 37.93, H 7.52, and N 4.02; Found: C 37.63, H 7.46, and N 4.06.

***S*** **-8-Bromooctyl methanesulfonothioate** (560 mg, 86%) was obtained from sodium methanethiosulfonate (288 mg, 2.15 mmol) and 1,8-dibromooctane (2.82 g, 10.37 mmol) following procedure **iv**. ^1^H NMR (CDCl_3_): 3.41 (t, *J* = 6.8 Hz, 2H), 3.33 (s, 3H), 3.17 (t, *J* = 7.4 Hz, 2H), 1.89–1.82 (m, 2H), 1.81–1.73 (m, 2H), and 1.48–1.32 (m, 8H); ^13^C NMR (CDCl_3_): 50.9, 36.7, 34.2, 32.9, 29.7, 29.0, 28.7, and 28.2.

***N*** **,** ***N*** **,** ***N*** **-Trimethyl-8-(methylsulfonylthio)octan-1-aminium bromide** (**AMTS8**, 236 mg, 76%) was obtained from trimethylamine (33% w/w solution in ethanol, 30 mL) and *S*-8-bromooctyl methanesulfonothioate (260 mg, 0.85 mmol) following procedure **vi**. ^1^H NMR (CDCl_3_): 3.67–3.62 (m, 2H), 3.45 (s, 9H), 3.37 (s, 3H), 3.19 (t, *J* = 7.4 Hz, 2H), 1.82–1.74 (m, 4H), and 1.48–1.34 (m, 8H); ^13^C NMR (CDCl_3_): 66.9, 53.6, 51.0, 36.6, 29.4, 29.0, 28.7, 28.3, 26.1, and 23.2.

***S*** **-9-Bromononyl methanesulfonothioate** (480 mg, 71%) was obtained from sodium methanethiosulfonate (268 mg, 2.14 mmol) and 1,9-dibromononane (2.86 g, 10.0 mmol) following procedure **iv**. ^1^H NMR (CDCl_3_): 3.41 (t, *J* = 6.8 Hz, 2H), 3.32 (s, 3H), 3.17 (t, *J* = 7.4 Hz, 2H), 1.89–1.82 (m, 2H), 1.80–1.73 (m, 2H), 1.42 (m, 6H), and 1.31 (m, 4H); ^13^C NMR (CDCl_3_): 50.9, 36.7, 34.3, 33.0, 29.7, 29.4, 29.1, 28.8, 28.7, and 28.3.

***N*** **,** ***N*** **,** ***N*** **-Trimethyl-9-(methylsulfonylthio)nonan-1-aminium bromide** (**AMTS9**, 212 mg, 66%) was obtained from trimethylamine (33% w/w solution in ethanol, 30 mL) and *S*-9-bromononyl methanesulfonothioate (270 mg, 0.85 mmol) following procedure **vi**. ^1^H NMR (CDCl_3_): 3.64–3.60 (m, 2H), 3.45 (s, 9H), 3.36 (s, 3H), 3.18 (t, *J* = 7.2 Hz, 2H), 1.81–1.73 (m, 4H), and 1.44–1.33 (m, 10H); ^13^C NMR (CDCl_3_): 67.0, 53.6, 50.9, 36.7, 29.5, 29.1, 28.8, 28.4, 26.2, and 23.3.

***N*** **,** ***N*** **,** ***N*** **-Trimethyl-10-(methylsulfonylthio)decan-1-aminium bromide** (**AMTS10**, 60 mg, 23%) was obtained from sodium methanethiosulfonate (103 mg, 0.77 mmol) and 10-bromo-*N*,*N*,*N*-trimethyldecan-1-aminium bromide (238 mg, 0.66 mmol) following procedure **v**. ^1^H NMR (DMSO-*d*_6_): 3.52 (s, 3H), 3.30–3.26 (m, 2H), 3.20 (t, *J* = 7.4 Hz, 2H), 3.05 (s, 9H), 1.74–1.62 (m, 4H), and 1.40–1.22 (m, 12H); ^13^C NMR (DMSO-*d*_6_): 65.9, 52.8, 50.8, 36.2, 29.5, 29.4, 29.1, 29.0, 28.5, 26.4, and 22.7.

***S*** **-11-Bromoundecyl methanesulfonothioate** (350 mg, 60%) was obtained from sodium methanethiosulfonate (228 mg, 1.70 mmol) and 1,11-dibromoundecane (3.3 g, 10.51 mmol) following procedure **iv**. ^1^H NMR (CDCl_3_): 3.41 (t, *J* = 6.8 Hz, 2H), 3.33 (s, 3H), 3.17 (t, *J* = 7.4 Hz, 2H), 1.89–1.82 (m, 2H), 1.80–1.73 (m, 2H), 1.41 (m, 4H), and 1.29 (m, 10H); ^13^C NMR (CDCl_3_): 50.9, 36.7, 34.4, 33.0, 29.7, 29.6, 29.2, 29.0, 28.8, and 28.4.

***N*** **,** ***N*** **,** ***N*** **-Trimethyl-11-(methylsulfonylthio)undecan-1-aminium bromide** (**AMTS11**, 130 mg, 41%) was obtained from trimethylamine (33% w/w solution in ethanol, 60 mL) and *S*-11-bromoundecyl methanesulfonothioate (270 mg, 0.78 mmol) following procedure **vi**. ^1^H NMR (CDCl_3_): 3.63–3.59 (m, 2H), 3.47 (s, 9H), 3.35 (s, 3H), 3.18 (t, *J* = 7.4 Hz, 2H), 1.80–1.73 (m, 4H), and 1.42–1.28 (m, 14H); ^13^C NMR (DMSO-*d*_6_): 65.9, 52.8, 50.8, 36.2, 29.5, 29.4, 29.2, 29.1, 28.6, 26.4, and 22.7.

***N*** **,** ***N*** **,** ***N*** **-Trimethyl-12-(methylsulfonylthio)dodecan-1-aminium bromide** (**AMTS12**, 47 mg, 13%) was obtained from sodium methanethiosulfonate (114 mg, 0.85 mmol) and 12-bromo-*N*,*N*,*N*-trimethyldodecan-1-aminium bromide (387 mg, 0.91 mmol) following procedure **v**. ^1^H NMR (CDCl_3_): 3.62–3.57 (m, 2H), 3.47 (s, 9H), 3.34 (s, 3H), 3.18 (t, *J* = 7.4 Hz, 2H), 1.81–1.71 (m, 4H), and 1.46–1.27 (m, 16H); ^13^C NMR (CDCl_3_): 67.3, 53.6, 50.9, 36.7, 29.6, 29.4, 29.0, 28.7, 26.3, and 23.4.

**13-Hydroxy-** ***N*** **,** ***N*** **,** ***N*** **-trimethyltridecan-1-aminium bromide** (1.33 g, 80%) was obtained from trimethylamine (33% w/w solution in ethanol, 60 mL) and 13-bromotridecan-1-ol (1.16 g, 4.15 mmol) following procedure **i**. ^1^H NMR (DMSO-*d*_6_): 4.34 (t, *J* = 5.1 Hz, 1H), 3.37 (m, 2H), 3.30–3.25 (m, 2H), 3.05 (s, 9H), 1.70–1.60 (m, 2H), 1.42–1.27 (m, 2H), and 1.27–1.20 (m, 18H); ^13^C NMR (DMSO-*d*_6_): 65.9, 61.4, 52.8, 33.2, 29.8, 29.7, 29.5, 29.2, 26.4, 26.2, and 22.7.

**13-Bromo-** ***N*** **,** ***N*** **,** ***N*** **-trimethyltridecan-1-aminium bromide** (610 mg, 71%) was obtained from hydrobromic acid (48% aqueous, 20 mL) and 13-hydroxy-*N*,*N*,*N*-trimethyltridecan-1-aminium bromide (728 mg, 2.15 mmol) following procedure **ii**. ^1^H NMR (DMSO-*d*_6_): 3.53 (t, *J* = 6.6 Hz, 2H), 3.29–3.24 (m, 2H), 3.04 (s, 9H), 1.82–1.75 (m, 2H), 1.66 (m, 2H), and 1.38–1.26 (m, 18H); ^13^C NMR (DMSO-*d*_6_): 65.9, 52.8, 36.0, 32.9, 29.7, 29.6, 29.5, 29.2, 28.8, 28.2, 26.4, and 22.7.

***N*** **,** ***N*** **,** ***N*** **-Trimethyl-13-(methylsulfonylthio)tridecan-1-aminium bromide** (**AMTS13**, 320 mg, 63%) was obtained from sodium methanethiosulfonate (200 mg, 1.49 mmol) and 13-bromo-*N*,*N*,*N*-trimethyltridecan-1-aminium bromide (470 mg, 1.17 mmol) following procedure **v**. ^1^H NMR (DMSO-*d*_6_): 3.49 (s, 3H), 3.27–3.23 (m, 2H), 3.17 (t, *J* = 7.4 Hz, 2H), 3.02 (s, 9H), 1.70–1.60 (m, 4H), and 1.36–1.20 (m, 18H); ^13^C NMR (DMSO-*d*_6_): 65.9, 52.8, 50.8, 36.2, 29.7, 29.6, 29.5, 29.2, 29.1, 28.6, 26.4, and 22.7; Anal. Calcd (%) for C_17_H_38_NO_2_S_2_ Br•H_2_O: C 45.32, H 8.95, and N 3.11; Found: C 45.28, H 8.83, and N 3.08.

***S*** **-14-Bromotetradecyl methanesulfonothioate** (340 mg, 88%) was obtained from sodium methanethiosulfonate (134 mg, 1.00 mmol) and 1,14-dibromotetradecane (1.00 g, 2.81 mmol) following procedure **iv**. ^1^H NMR (CDCl_3_): 3.41 (t, *J* = 6.8 Hz, 2H), 3.23 (s, 3H), 3.17 (t, *J* = 7.4 Hz, 2H), 1.89–1.82 (m, 2H), 1.80–1.73 (m, 2H), 1.42 (m, 4H), and 1.26 (m, 16H); ^13^C NMR (CDCl_3_): 50.9, 36.7, 34.4, 33.1, 29.8, 29.7, 29.6, 29.2, 29.0, 28.8, and 28.4.

***N*** **,** ***N*** **,** ***N*** **-Trimethyl-14-(methylsulfonylthio)tetradecan-1-aminium bromide** (**AMTS14**, 120 mg, 35%) was obtained from trimethylamine (33% w/w solution in ethanol, 25 mL) and *S*-14-bromotetradecyl methanesulfonothioate (300 mg, 0.77 mmol) following procedure **vi**. ^1^H NMR (DMSO-*d*_6_): 3.49 (s, 3H), 3.29–3.24 (m, 2H), 3.17 (t, *J* = 7.4 Hz, 2H), 3.03 (s, 9H), 1.70–1.59 (m, 4H), and 1.36–1.20 (m, 20H); ^13^C NMR (DMSO-*d*_6_): 65.9, 52.8, 50.8, 36.2, 29.7, 29.6, 29.5, 29.2, 29.1, 28.6, 26.4, and 22.7.

**15-Bromo-** ***N*** **,** ***N*** **,** ***N*** **-trimethylpentadecan-1-aminium bromide** (930 mg, 91%) was obtained from hydrobromic acid (48% aqueous, 20 mL) and 15-hydroxy-*N*,*N*,*N*-trimethylpentadecan-1-aminium bromide (877 mg, 2.39 mmol) following procedure **ii**. ^1^H NMR (CDCl_3_): 3.61–3.57 (m, 2H), 3.48 (s, 9H), 3.42 (t, *J* = 6.8 Hz, 2H), 1.89–1.82 (m, 2H), 1.79–1.70 (m, 2H), and 1.46–1.23 (m, 22H); ^13^C NMR (CDCl_3_): 67.2, 53.6, 34.4, 33.0, 29.8, 29.7, 29.6, 29.5, 29.0, 28.4, 26.4, and 23.4.

***N*** **,** ***N*** **,** ***N*** **-Trimethyl-15-(methylsulfonylthio)pentadecan-1-aminium bromide** (**AMTS15**, 180 mg, 20%) was obtained from sodium methanethiosulfonate (281 mg, 2.09 mmol) and 15-bromo-*N*,*N*,*N*-trimethylpentadecan-1-aminium bromide (827 mg, 1.93 mmol) following procedure **v**. ^1^H NMR (DMSO-*d*_6_): 3.49 (s, 3H), 3.26–3.22 (m, 2H), 3.17 (t, *J* = 7.2 Hz, 2H), 3.02 (s, 9H), 1.70–1.59 (m, 4H), and 1.37–1.20 (m, 22H); ^13^C NMR (DMSO-*d*_6_): 65.9, 52.8, 50.8, 36.2, 29.7, 29.6, 29.5, 29.2, 29.1, 28.6, 26.4, and 22.7.

**16-Bromo-** ***N*** **,** ***N*** **,** ***N*** **-trimethylhexadecan-1-aminium bromide** (800 mg, 84%) was obtained hydrobromic acid (48% aqueous, 20 mL) and 16-hydroxy-*N*,*N*,*N*-trimethylhexadecan-1-aminium bromide (820 mg, 2.15 mmol) following procedure **ii**. ^1^H NMR (CDCl_3_): 3.62–3.57 (m, 2H), 3.48 (s, 9H), 3.41 (t, *J* = 6.8 Hz, 2H), 1.89–1.82 (m, 2H), 1.79–1.71 (m, 2H), and 1.46–1.22 (m, 24H); ^13^C NMR (CDCl_3_): 67.2, 53.6, 34.4, 33.0, 29.8, 29.7, 29.6, 29.5, 29.0, 28.4, 26.4, and 23.4.

***N*** **,** ***N*** **,** ***N*** **-Trimethyl-16-(methylsulfonylthio)hexadecan-1-aminium bromide** (**AMTS16**, 120 mg, 21%) was obtained from sodium methanethiosulfonate (160 mg, 1.20 mmol) and 16-bromo-*N*,*N*,*N*-trimethylhexadecan-1-aminium bromide (564 mg, 1.27 mmol) following procedure **v**. ^1^H NMR (CDCl_3_): 3.62–3.58 (m, 2H), 3.47 (s, 9H), 3.34 (s, 3H), 3.18 (t, *J* = 7.4 Hz, 2H), 1.80–1.70 (m, 4H), and 1.46–1.22 (m, 24H); ^13^C NMR (CDCl_3_): 67.1, 53.6, 50.9, 36.8, 29.8, 29.7, 29.6, 29.5, 29.4, 29.1, 28.8, 26.4, and 23.4.

***S*** **-17-Bromoheptadecyl methanesulfonothioate** (36 mg, 93%) was obtained from 1,17-dibromoheptadecane (113 mg, 0.28 mmol) and sodium methanethiosulfate (12 mg, 0.09 mmol) following the general procedure **iv**. ^1^H NMR (CDCl_3_): 3.41 (t, *J* = 6.8 Hz, 2H), 3.32 (s, 3H), 3.17 (t, *J* = 7.4 Hz, 2H), 1.88–1.81 (m, 2H), 1.79–1.72 (m, 2H), and 1.42–1.20 (m, 26H); ^13^C NMR (CDCl_3_): 50.9, 36.8, 34.4, 33.1, 29.9, 29.8, 29.7, 29.6, 29.2, 29.0, 28.8, and 28.4.

***N*** **,** ***N*** **,** ***N*** **-Trimethyl-17-(methylsulfonylthio)heptadecan-1-aminium bromide** (**AMTS17**, 5 mg, 12%) was obtained from trimethylamine (33% w/w solution in ethanol, 2.6 mL) and *S*-17-bromoheptadecyl methanesulfonothioate (34 mg, 0.08 mmol) following general procedure **vi**. ^1^H NMR (CDCl_3_): 3.59–3.56 (m, 2H), 3.47 (s, 9H), 3.33 (s, 3H), 3.17 (t, *J* = 7.4 Hz, 2H), 1.80–1.71 (m, 4H), and 1.46–1.22 (m, 26H); ^13^C NMR (CDCl_3_): 67.3, 53.6, 50.9, 36.8, 29.8, 29.7, 29.6, 29.4, 29.2, 28.8, 26.4, and 23.4; Anal. Calcd (%) for C_21_H_46_NO_2_S_2_ Br•H_2_O: C 49.78, H 9.55, and N 2.76; Found: C 49.41, H 9.57, and N 2.75.

***S*** **-18-Bromooctadecyl methanesulfonothioate** (256 mg, 63%) was obtained from 1,18-dibromooctadecane (1.143 g, 2.77 mmol) and sodium methanethiosulfate (123 mg, 0.92 mmol) following procedure **iv**. ^1^H NMR (CDCl_3_): 3.41 (t, *J* = 6.8 Hz, 2H), 3.32 (s, 3H), 3.17 (t, *J* = 7.2 Hz, 2H), 1.89–1.82 (m, 2H), 1.80–1.73 (m, 2H), and 1.42–1.26 (m, 28H); ^13^C NMR (CDCl_3_): 50.9, 36.7, 34.4, 33.1, 29.9, 29.8, 29.7, 29.6, 29.2, 29.0, 28.8, and 28.4.

***N*** **,** ***N*** **,** ***N*** **-Trimethyl-18-(methylsulfonylthio)octadecan-1-aminium bromide** (**AMTS18**, 223 mg, 93%) was obtained from trimethylamine (33% w/w solution in ethanol, 14 mL) and *S*-18-bromooctadecyl methanesulfonothioate (211 mg, 0.48 mmol) following procedure **vi**. ^1^H NMR (CDCl_3_): 3.60–3.56 (m, 2H), 3.48 (s, 9H), 3.33 (s, 3H), 3.17 (t, *J* = 7.4 Hz, 2H), 1.80–1.71 (m, 4H), and 1.43–1.22 (m, 28H); ^13^C NMR (CDCl_3_): 67.2, 53.6, 50.9, 36.8, 29.8, 29.7, 29.6, 29.5, 29.2, 28.8, 26.4, and 23.4.

***S*** **-19-Bromononadecyl methanesulfonothioate** (64 mg, 96%) was obtained from 1,19-dibromononadecane (179 mg, 0.42 mmol) and sodium methanethiosulfate (19 mg, 0.14 mmol) following general procedure **iv**. ^1^H NMR (CDCl_3_): 3.40 (t, *J* = 6.8 Hz, 2H), 3.31 (s, 3H), 3.16 (t, *J* = 7.4 Hz, 2H), 1.88–1.81 (m, 2H), 1.79–1.72 (m, 2H) and 1.41–1.22 (m, 30H); ^13^C NMR (CDCl_3_): 50.9, 36.7, 34.4, 33.1, 29.9, 29.8, 29.7, 29.6, 29.2, 29.0, 28.8, and 28.4.

***N*** **,** ***N*** **,** ***N*** **-Trimethyl-19-(methylsulfonylthio)nonadecan-1-aminium bromide** (**AMTS19**, 26 mg, 34%) was obtained from trimethylamine (33% w/w solution in ethanol, 25 mL) and *S*-19-bromononadecyl methanesulfonothioate (68 mg, 0.15 mmol) following general procedure **vi**. ^1^H NMR (CDCl_3_): 3.60–3.55 (m, 2H), 3.47 (s, 9H), 3.33 (s, 3H), 3.17 (t, *J* = 7.4 Hz, 2H), 1.80–1.70 (m, 4H), and 1.46–1.22 (m, 30H); ^13^C NMR (CDCl_3_): 67.4, 53.7, 50.9, 36.8, 29.9, 29.8, 29.7, 29.6, 29.4, 29.2, 28.8, 26.4, and 23.4.

***S*** **-20-Bromoicosyl methanesulfonothioate** (219 mg, 62%) was obtained from 1,20-dibromoicosane (1.01 g, 2.29 mmol) and sodium methanethiosulfate (103 mg, 0.76 mmol) following general procedure **iv**. ^1^H NMR (CDCl_3_): 3.40 (t, *J* = 6.8 Hz, 2H), 3.32 (s, 3H), 3.16 (t, *J* = 7.4 Hz, 2H), 1.88–1.81 (m, 2 H), 1.79–1.72 (m, 2H), and 1.42–1.25 (m, 32H); ^13^C NMR (CDCl_3_): 50.9, 36.7, 34.4, 33.1, 29.9, 29.8, 29.7, 29.6, 29.2, 29.0, 28.8, and 28.4.

***N*** **,** ***N*** **,** ***N*** **-Trimethyl-20-(methylsulfonylthio)icosan-1-aminium bromide** (**AMTS20**, 117 mg, 82%) was obtained from trimethylamine (33% w/w solution in ethanol, 10 mL) and *S*-20-bromoicosyl methanesulfonothioate (129 mg, 0.27 mmol) following general procedure **vi**. ^1^H NMR (CDCl_3_): 3.60–3.56 (m, 2H), 3.48 (s, 9H), 3.33 (s, 3H), 3.17 (t, *J* = 7.2 Hz, 2H), 1.80–1.73 (m, 4H), and 1.46–1.24 (m, 32H); ^13^C NMR (CDCl_3_): 67.4, 53.6, 50.9, 36.8, 29.9, 29.8, 29.7, 29.6, 29.4, 29.2, 28.8, 26.4, and 23.4.

**13-[Methoxythio(** ***d*** **_3_)]-** ***N*** **,** ***N*** **,** ***N*** **-trimethyltridecan-1-aminium bromide**. To a 5-mm NMR tube containing 0.6 mL of CD_3_OD was added 8.6 mg of Na metal (623 µM of CD_3_ONa, 0.373 mmol). Sodium metal was allowed to dissolve completely and the NMR tube was kept at room temperature for one hour. **AMTS13** (5 mg, 0.011 mmol) was then added in one portion to the NMR tube. Soon after the addition ^1^H NMR spectrum of the resultant mixture was acquired and showed complete disappearance of the starting material. The spectrum for the crude product is as follows:

^1^H NMR (CD_3_OD) δ 3.30–3.44 (m, 2H), 3.12 (s, 9H), 2.66 (t, *J* = 7.2 Hz, 2H), 1.78 (m, 2H), 1.66 (m, 2H), and 1.30–1.40 (m, 18H); ^13^C NMR (CD_3_OD) δ 66.6, 62.2, 52.3, 48.6, 38.5, 29.5, 29.4, 29.3, 29.1, 29.0, 28.2, 26.2, and 22.8.

### Measurement of AChE inhibition

Greenbugs, soybean aphids, or fruit flies were stored frozen at −20°C, as were crude preparations of washed human red blood cell membranes prepared as described elsewhere [47]. For experiments involving exposure to test compounds and subsequent dialysis, the insect samples were homogenized twice for 10 seconds each in ground glass homogenizers containing 100 volumes of ice-cold 0.09% NaCl, 0.1 M sodium phosphate (pH 7.4) and 0.1% BSA. RBC samples were prepared similarly but by sonication for 5 to 10 sec in buffer with added Triton X-100 (0.5% v/v). In both cases the resulting fine suspensions were diluted 30-fold in the homogenization buffer before treatments and assays, and they were thoroughly re-suspended by vortex mixing as each aliquot was transferred to the reaction tubes. Strict attention to this procedure resulted in duplicate agreements within ±2% for the greenbug AChE activity and within ±1% for RBC AChE activity.

For preliminary experiments involving removal of test compounds by size-exclusion chromatography, insect and red cell samples were homogenized for 2 min in 0.1 M sodium phosphate (pH 7.4) and centrifuged at 15,000×g for 15 min to obtain supernatants for inhibitor exposure or enzyme assay. In certain experiments, 0.3% (v/v) Triton X-100 was added to the buffer in order to solubilize the enzyme (extraction efficiency 85–90%). This procedure did not increase total AChE activity or alter the percentage of inhibition by the methanethiosulfonate agents tested.

Experiments calling for sample exposure to inhibitors or reactivating reagents typically involved one or more steps to separate the small molecules from the enzyme before determinations of AChE activity. In the initial phases of the study, these steps utilized Centricon Spin Columns containing Sepharose G-10 size-exclusion gel-filtration resin, which provided very rapid separation during 10 min of centrifugation at 1000×g. This procedure, followed by 50-fold dilution before assay (with 0.1 M sodium phosphate buffer, pH 7.4), enhanced throughput for screening purposes. Final data reported in this article, however, were obtained after dialysis for 16–24 hr against two changes of the buffer in more than 100-fold excess. For present purposes this standard biochemical procedure was validated by control experiments demonstrating its ability to remove all traces of inhibitory activity as tested with sentinel samples of AChE.

The AChE assays were based on an established radiometric technique in which product (^3^H-labeled acetic acid) liberated enzymatically from substrate (^3^H-labeled acetylcholine, 50 nCi in a final reaction volume of 100 µL at pH 7.4) is partitioned into 4 mL of toluene-isoamyl alcohol (5∶1, v/v) with scintillation fluor [48], [49]. As a rule, the assays were performed with substrate in a concentration of 0.1 µM. This condition allowed maximum sensitivity (active samples more than 10 times the buffer-only blanks) with small samples (500 µg wet weight equivalent) and high temporal resolution (assay times as short as 5 min). Also, because of the low substrate concentrations a reversible 50% inhibition was expected to occur at concentrations near the true *K*_i_. When necessary, substrate concentration was adjusted by diluting stock material (99 mCi/mM) with unlabelled acetylcholine chloride. Assay duration, at room temperature, was rigorously controlled to ensure that signal was robust (>5 times blank value) and remained linear with respect to time and amount of sample present (typical conditions, 5 min for concentrated samples or up to 4 hours for highly dilute or low activity samples).

### AChE Reactivation by β-Mercaptoethanol

Reactivation by BME was examined with AChE samples that were exposed for 1 hr to an inhibitor at 6.0 µM final concentration, followed by dialysis overnight against two changes of >100 volumes of 0.1 M sodium phosphate buffer at pH 7.4. This treatment led to 99% inhibition of the greenbug enzyme and 43% inhibition of the red cell enzyme. Treated samples were exposed to BME at a final concentration of 100 mM for 0.5, 1 or 2 hr, followed by a second overnight dialysis against the phosphate buffer.

### Multiple Molecular Dynamics Simulations of AChE Complexes

Multiple molecular dynamics simulations (MMDSs) were performed using the PMEMD module of the AMBER 8.0 program [50] with a revised force field (to be published) that is based on the second-generation AMBER force field (frcmod.ff99SB) [51]. The topology and coordinate files were generated by the PREP, LINK, EDIT, and PARM modules of the AMBER 5.0 program [50]. All simulations used (1) a dielectric constant of 1.0, (2) the Berendsen coupling algorithm [52], (3) a periodic boundary condition at a constant temperature of 300 K and a constant pressure of 1 atm with isotropic molecule-based scaling, (4) the Particle Mesh Ewald method to calculate long-range electrostatic interactions [53], (5) a time step of 1.0 fs, (6) the SHAKE-bond-length constraints applied to all the bonds involving the H atom, (7) saving the image closest to the middle of the “primary box” to the restart and trajectory files, (8) unformatted restart file, and (9) default values of all other inputs of the PMEMD module. All simulations were performed on eight Apple Mac Pros each equipped with eight Intel Woodcrest cores at a clock rate of 3.0 GHz.

The atomic charges of **AMTS13** and **AMTS17** were obtained according to the RESP procedure [54] with *ab initio* calculations at the HF/6-31G*//HF/6-31G* level using the Gaussian98 program [55]. The human and greenbug AChE structures were taken from the coordinate files with Protein Data Bank codes of 1B41 [17] and 2HCP [9], respectively. For the human enzyme, His447 and His284 were treated as HID; His223 and His387 were treated as HIE; all other His residues were treated as HIP. For the greenbug AChE, His45, His411, His442, and His500 were treated as HID; all other His residues were treated as HIP. The initial structure of the inhibitor-bound AChE was generated by manually inserting **AMTS13** or **AMTS17** in its fully extended conformation into the active site of the respective AChE that was devoid of fasciculin, acetylcholine, water molecules, and ions. The methanethiosulfonate and aminium moieties of **AMTS13** and **AMTS17** were placed at the bottom of the active site of the human and greenbug AChEs, respectively. The resulting complexes were refined by a two-step energy minimization. Step 1 used (1) a positional constraint applied to AChE (IBELLY = 1), (2) 100 cycles of steepest-descent minimization followed by 400 cycles of conjugate-gradient minimization, and (3) a dielectric constant of 1.0. Step 2 used (1) no positional constraint (IBELLY = 0), (2) 10 cycles of steepest-descent minimization followed by 490 cycles of conjugate-gradient minimization, and (3) a dielectric constant of 1.0.

The energy-minimized AChE complexes were solvated with 14,357 and 17,549 TIP3P water molecules [56], respectively (EDIT input: 216000 water molecules, 0.4170 for the charge on the water hydrogen atom, removing any water molecule whose oxygen atom is closer than 2.2 Å to any solute atom, removing any water molecule whose hydrogen atom is closer than 2.0 Å to any solute atom, and removing any water molecule that is farther than 8.2 Å along the x-, y-, and z-axes from any atom of solute). The solvated human and insect AChE complex systems had a total of 51,288 and 61,205 atoms; they were first energy-minimized for 200 steps to remove close van der Waals contacts in the system, slowly heated to 300 K (10 K/ps under constant temperature and volume). Thirteen 10-ns-long simulations (each with a unique seed number for starting velocities) were carried out for the human AChE complex whereas four 10-ns-long simulations were carried out for the insect complex.

For each simulation, a time-average structure was obtained from 1000 trajectories collected at 1.0-ps intervals during the last 1-ns period using the CARNAL module of AMBER 5.0. For the 13 simulations of the human complex, the distances of the Ser203 hydroxyl oxygen atom to the **AMTS13** thiol sulfur atom calculated from the initial structure and the 13 time-average structures were 4.1, 6.2, 6.4, 9.6, 18.9, 8.5, 5.8, 13.5, 8.7, 8.9, 10.4, 11.0, 4.7, and 8.1 Å, respectively, indicating good sampling achieved by the 10-ns-long MMDSs. With water molecules stripped off, the average structure with the shortest distance was then energy minimized (performing 50 cycles of steepest-descent minimization and then 150 cycles of conjugate-gradient minimization using a dielectric constant of 80.0). The energy-minimized average structure was chosen as the MMDS-refined 3D model. Likewise, for the greenbug AChE complex, the distance between the Cys289 sulfur atom and the **AMTS17** thiol sulfur atom was used to generate the MMDS-refined 3D model, and the distances calculated from the initial structure and the four time-average structures were 6.5, 3.8, 7.5, 5.5, and 8.9 Å, respectively. The coordinates of the initial and the MMDS-refined 3D models of the human and greenbug AChE complexes are provided in Datasets S1, S2, S3, S4 of Supporting Information, respectively.

## Supporting Information

Dataset S1 The initial model of the human AChE in complex with AMTS13
(0.23 MB TXT)

Dataset S2 The refined model of the human AChE in complex with AMTS13
(0.23 MB TXT)

Dataset S3 The initial model of the greenbug AChE in complex with AMTS17
(0.24 MB TXT)

Dataset S4 The refined model of the greenbug AChE in complex with AMTS17
(0.24 MB TXT)

## References

[1] Lazar MD, Michel GJ, Weng Y, Wang WC, Porter KB (1999). Development of greenbug resistance in wheat;.

[2] Ragsdale DW, McCornack BP, Venette RC, Potter BD, Macrae IV (2007). Economic threshold for soybean aphid (Hemiptera: Aphididae).. J Econ Entomol.

[3] McCornack BP, Ragsdale DW, Venette RC (2004). Demography of soybean aphid (Homoptera: Aphididae) at summer temperatures.. J Econ Entomol.

[4] Taylor P, Radic Z (1994). The cholinesterases: from genes to proteins.. Annu Rev Pharmacol Toxicol.

[5] Lewis G, Thompson H, Smagghe G (2007). In focus: pesticides and honeybees – the work of the ICP-BR Bee Protection Group.. Pest Manag Sci.

[6] Fialka JJ (2006). EPA scientists cite pressure in pesticide study..

[7] Pezzementi L, Rowland M, Wolfe M, Tsigelny I (2006). Inactivation of an invertebrate acetylcholinesterase by sulfhydryl reagents: the roles of two cysteines in the catalytic gorge of the enzyme.. Invert Neurosci.

[8] Pang Y-P (2006). Novel acetylcholinesterase target site for malaria mosquito control.. PLoS ONE.

[9] Pang Y-P (2007). Species marker for developing novel and safe pesticides.. Bioorg Med Chem Lett.

[10] Randall WR, Rimer M, Gough NR (1994). Cloning and analysis of chicken acetylcholinesterase transcripts from muscle and brain.. Biochim Biophys Acta.

[11] Gao JR, Kambhampati S, Zhu KY (2002). Molecular cloning and characterization of a greenbug (*Schizaphis graminum*) cDNA encoding acetylcholinesterase possibly evolved from a duplicate gene lineage.. Insect Biochem Mol Biol.

[12] Zahavi M, Tahori AS, Klimer F (1972). An acetylcholinesterase sensitive to sulfhydryl inhibitors.. Biochim Biophys Acta.

[13] Smissaert HR (1976). Reactivity of a critical sulfhydryl group of the acetylcholinesterase from aphids (*Myzus persicae*).. Pest Biochem Physiol.

[14] Manulis S, Ishaaya I, Perry AS (1981). Acetylcholinesterase of *Aphis citricola*: properties and significance in determining toxicity of systemic organophosphorus and carbamates compounds.. Pest Biochem Physiol.

[15] Brestkin AP, Maizel EB, Moralev SN, Novozhilov KV, Sazonova IN (1985). Cholinesterases of aphids. I. Isolation, partial purification and some properties of cholinesterases from spring grain aphid *Schizaphis gramina* (Rond.).. Insect Biochem.

[16] Novoshilov KV, Brestkin AP, Khovanskikh AE, Maizel EB, Moralev SN (1989). Cholinesterases of aphids. III. Sensitivity of acetylcholinesterases to several inhibitors as a possible phylogenetic character.. Insect Biochem.

[17] Kryger G, Harel M, Giles K, Toker L, Velan B (2000). Structures of recombinant native and E202Q mutant human acetylcholinesterase complexed with the snake-venom toxin fasciculin-II.. Acta Crystallogr Sect D.

[18] Zauhar RJ, Colbert CL, Morgan RS, Welsh WJ (2000). Evidence for a strong sulfur-aromatic interaction derived from crystallographic data.. Biopolymers.

[19] Erlanson DA, Braisted AC, Raphael DR, Randal M, Stroud RM (2000). Site-directed ligand discovery.. Proc Natl Acad Sci USA.

[20] Pang Y-P, Xu K, Kollmeyer TM, Perola E, McGrath WJ (2001). Discovery of a new inhibitor lead of adenovirus proteinase: steps toward selective, irreversible inhibitors of cysteine proteinases.. FEBS Lett.

[21] Johnson JL, Cusack B, Hughes TF, McCullough EH, Fauq A (2003). Inhibitors tethered near the acetylcholinesterase active site serve as molecular rulers of the peripheral and acylation sites.. J Biol Chem.

[22] Boyd AE, Marnett AB, Wong L, Taylor P (2000). Probing the active center gorge of acetylcholinesterase by fluorophores linked to substituted cysteines.. J Biol Chem.

[23] Adams MD, Celniker SE, Holt RA, Evans CA, Gocayne JD (2000). The genome sequence of *Drosophila melanogaster*.. Science.

[24] Mutero A, Pralavorio M, Bride JM, Fournier D (1994). Resistance-associated point mutations in insecticide-insensitive acetylcholinesterase.. Proc Natl Acad Sci USA.

[25] Kono Y, Tomita T (2006). Amino acid substitutions conferring insecticide insensitivity in Ace-paralogous acetylcholinesterase.. Pestic Biochem Physiol.

[26] Malcolm CA, Bourguet D, Ascolillo A, Rooker SJ, Garvey CF (1998). A sex-linked Ace gene, not linked to insensitive acetylcholinesterase-mediated insecticide resistance in *Culex pipiens*.. Insect Mol Biol.

[27] Tomita T, Hidoh O, Kono Y (2000). Absence of protein polymorphism attributable to insecticide-insensitivity of acetylcholinesterase in the green rice leafhopper, *Nephotettix cincticeps*.. Insect Biochem Mol Biol.

[28] Gao J-R, Zhu KY (2002). Increased expression of an acetylcholinesterase gene may confer organophosphate resistance in the greenbug, *Schizaphis graminum* (Homoptera: Aphididae).. Pestic Biochem Physiol.

[29] Weill M, Fort P, Berthomieu A, Dubois MP, Pasteur N (2002). A novel acetylcholinesterase gene in mosquitoes codes for the insecticide target and is non-homologous to the ace gene in *Drosophila*.. Proc Biol Sci.

[30] Baek JH, Kim JI, Lee D-W, Chung BK, Miyata T (2005). Identification and characterization of ace1-type acetylcholinesterase likely associated with organophosphate resistance in *Plutella xylostella*.. Pestic Biochem Physiol.

[31] Mamiya A, Ishikawa Y, Kono Y (1997). Acetylcholinesterase in insecticide resistant *Culex tritaeniorhynchus*: characteristics accompanying insensitivity to inhibitors.. Appl Entomol Zool.

[32] Sussman JL, Harel M, Frolow F, Oefner C, Goldman A (1991). Atomic structure of acetylcholinesterase from *Torpedo californica*: a prototypic acetylcholine-binding protein.. Science.

[33] Raves ML, Harel M, Pang YP, Silman I, Kozikowski AP (1997). Structure of acetylcholinesterase complexed with the nootropic alkaloid, (-)-huperzine A.. Nat Struct Biol.

[34] Smith DJ, Kenyon GL (1974). Nonessentiality of the active sulfhydryl group of rabbit muscle creatine kinase.. J Biol Chem.

[35] Javitch JA, Li X, Kaback J, Karlin A (1994). A cysteine residue in the third membrane-spanning segment of the human D2 dopamine receptor is exposed in the binding-site crevice.. Proc Natl Acad Sci USA.

[36] Chen JG, Liu-Chen S, Rudnick G (1997). External cysteine residues in the serotonin transporter.. Biochemistry.

[37] Zhang Y, Kanner BI (1999). Two serine residues of the glutamate transporter GLT-1 are crucial for coupling the fluxes of sodium and the neurotransmitter.. Proc Natl Acad Sci USA.

[38] Soreq H, Ben-Aziz R, Prody CA, Seidman S, Gnatt A (1990). Molecular cloning and construction of the coding region for human acetylcholinesterase reveals a G+C-rich attenuating structure.. Proc Natl Acad Sci USA.

[39] Kronman C, Velan B, Gozes Y, Leitner M, Flashner Y (1992). Production and secretion of high levels of recombinant human acetylcholinesterase in cultured cell lines: microheterogeneity of the catalytic subunit.. Gene.

[40] Rosenberry TL, Scoggin DM (1984). Structure of human erythrocyte acetylcholinesterase. Characterization of intersubunit disulfide bonding and detergent interaction.. J Biol Chem.

[41] Moore TL, O'Connor DE (1966). The reaction of methanesulfenyl chloride with alkoxides and alcohols: preparation of aliphatic sulfenate and sulfinate esters.. J Org Chem.

[42] Mohr W, Horn CR, Stahl J, Gladysz JA (2003). Convenient and convergent syntheses of long-chain α,ω-dibromides and diphosphines of the formula X(CH_2_)_n_X (n = 18–32).. Synthesis.

[43] Weber ME, Schlesinger PH, Gokel GW (2005). Dynamic assessment of bilayer thickness by varying phospholipid and hydraphile synthetic channel lengths.. J Am Chem Soc.

[44] Grayson EJ, Ward SJ, Hall AL, Rendle PM, Gamblin DP (2005). Glycosyl disulfides: novel glycosylating reagents with flexible aglycon alteration.. J Org Chem.

[45] Mary A, Renko DZ, Guillou C, Thal C (1998). Potent acetyl cholinesterase inhibitors: design, synthesis, and structure-activity relationships of bis-interacting ligands in the galanthamine series.. Bioorg Med Chem.

[46] Davey TW, Hayman AR (1998). Synthesis of ω-hydroxy quaternary ammonium bolaform surfactants.. Aust J Chem.

[47] Hammond PI, Kern C, Hong F, Kollmeyer TM, Pang Y-P (2003). Cholinesterase reactivation in vivo with a novel bis-oxime optimized by computer-aided design.. J Pharmacol Exp Ther.

[48] Brimijoin S (1979). Axonal transport and subcellular distribution of molecular forms of acetylcholinesterase in rabbit sciatic nerve.. Mol Pharmacol.

[49] Johnson CD, Russell RL (1975). A rapid simple radiometric assay for cholinesterase, suitable for multiple determinations.. Anal Biochem.

[50] Pearlman DA, Case DA, Caldwell JW, Ross WS, Cheatham TE (1995). AMBER, a package of computer programs for applying molecular mechanics, normal mode analysis, molecular dynamics and free energy calculations to simulate the structural and energetic properties of molecules.. Comput Phys Commun.

[51] Cornell WD, Cieplak P, Bayly CI, Gould IR, Merz KM (1995). A second generation force field for the simulation of proteins, nucleic acids, and organic molecules.. J Am Chem Soc.

[52] Berendsen HJC, Postma JPM, van Gunsteren WF, Di Nola A, Haak JR (1984). Molecular dynamics with coupling to an external bath.. J Chem Phys.

[53] Darden TA, York DM, Pedersen LG (1993). Particle Mesh Ewald: An N log(N) method for Ewald sums in large systems.. J Chem Phys.

[54] Cieplak P, Cornell WD, Bayly C, Kollman PA (1995). Application of the multimolecule and multiconformational resp methodology to biopolymers: charge derivation for DNA, RNA, and proteins.. J Comput Chem.

[55] Frisch MJ, Trucks GW, Schlegel HB, Gill PMW, Johnson BG (1999). GAUSSIAN 98, Revision A.7.

[56] Jorgensen WL, Chandreskhar J, Madura JD, Impey RW, Klein ML (1982). Comparison of simple potential functions for simulating liquid water.. J Chem Phys.

